# Caregiver-Provider Communication and Unmet Care Needs Among Older Adults: Evidence From NSOC and NHATS

**DOI:** 10.1093/geroni/igaf122.2156

**Published:** 2025-12-31

**Authors:** Jiaming Liang, Rafael Samper-Ternent

**Affiliations:** UTHealth Houston, San Antonio, Texas, United States; University of Texas Health Science Center at Houston, Houston, Texas, United States

## Abstract

Effective caregiver-provider communication is crucial for high-quality care in older adults, yet little is known about factors influencing communication quality (CQ) and its relationship with unmet care needs. Using data from the 2021–2023 National Study of Caregiving (NSOC) and the National Health and Aging Trends Study (NHATS), this study examined caregiver (N = 3,673) and care recipient (N = 2,439) characteristics associated with CQ and its impact on unmet care needs. CQ was assessed using three items: (1) whether the doctor listened to the caregiver, (2) whether the doctor checked if the caregiver understood, and (3) whether the doctor inquired about the caregiver’s need for help. Unmet care needs were categorized as none, one, or two or more. A mixed-effects model identified factors associated with CQ, and ordered logistic regression examined its association with unmet care needs among older adults. Higher CQ was reported among caregivers with frequent provider communication, Black and Hispanic caregivers (vs. non-Hispanic White), adult children caregivers (vs. spouses), those caring for older adults with dementia, and those with family support and social service use. Lower CQ was observed among caregivers aged 75 + (vs. < 55), those with higher education, and those reporting depression and anxiety. Higher CQ was associated with lower odds of unmet care needs (OR = 0.92, p < 0.001). Findings highlight the need to improve caregiver-provider communication, particularly for caregivers lacking family support and social resources or experiencing mental health difficulties, to reduce unmet care needs and improve care outcomes for older adults.

